# Design of an Integrated Near-Infrared Spectroscopy Module for Sugar Content Estimation of Apples

**DOI:** 10.3390/mi13040519

**Published:** 2022-03-26

**Authors:** Sangjin Byun

**Affiliations:** Division of Electronics and Electrical Engineering, Dongguk University, Seoul 04620, Korea; sjbyun@dongguk.edu; Tel.: +82-2-2260-3331

**Keywords:** NIR spectroscopy, sugar content estimation, CMOS integrated circuits

## Abstract

An integrated near-infrared (NIR) spectroscopy prototype module for sugar content estimation of apples is presented. Since this is the first attempt to design an integrated NIR spectroscopy module, we followed the design process as follows. First, we estimated the sugar content of apples using a tungsten halogen light source and a 700 nm–1000 nm NIR spectrometer with a 10 nm wavelength resolution and a 16b analog-to-digital converter (ADC) resolution. Second, we determined the most effective wavelengths among 31 evenly distributed wavelengths while observing the correlation coefficient, R^2^, and then we reduced the ADC resolution 1b by 1b starting from 16b while also observing the R^2^. Lastly, we designed an integrated NIR spectroscopy module with the selected eight wavelengths and a 13 ADC resolution. The module implemented in a 0.18 μm 1P6M CMOS process occupies a die area of 0.84 mm^2^. By utilizing this module with eight off-chip light emitting diodes (LED) and one photo diode (PD), the measured R^2^ and the standard error of calibration (SEC) were 0.365 and 0.686 brix, respectively.

## 1. Introduction

Apples are generally known to be composed of roughly 86% water; 12% sugars such as glucose, sucrose, and fructose; and an extremely small amount of cellulose, organic acids, fatty acids, amino acids, and a few kinds of minerals [1,2]. The quality of apples is determined by their sugar content, acidity, and firmness. Among these, the sugar content is the most important attribute that directly determines the taste of apples. The sugar content can be measured directly or estimated indirectly. Because the direct method measures the sugar content by using a digital refractometer after squeezing, it causes losses of apples and takes relatively long time. Moreover, since it is a sample investigation, it cannot guarantee the measured sugar content value for the other unmeasured apples. However, the indirect method estimates the sugar content by using a near-infrared (NIR) spectrometer measuring the intensities of the absorbed and reflected NIR lights from apples. Thus, we can quickly carry out a total investigation without any losses of apples.

In this paper, we designed an integrated NIR spectroscopy prototype module for indirect and nondestructive sugar content estimation of apples. Since this is the first attempt to design an integrated NIR spectroscopy module, we followed the design process as follows. First, we estimated the sugar content of apples using a tungsten halogen light source and a 700 nm–1000 nm NIR spectrometer with a 10 nm wavelength resolution and a 16b analog to digital converter (ADC) resolution. Second, to find the minimum requirements for the design of an integrated NIR spectroscopy module, we sorted out the most effective wavelengths from 31 while observing the correlation coefficient, R^2^, and then we reduced the ADC resolution 1b by 1b starting from 16b while also observing the R^2^. Lastly, we designed an integrated NIR spectroscopy module with the selected eight wavelengths and a 13b ADC resolution.

The integrated NIR spectroscopy module, which was implemented in a 0.18 μm 1P6M CMOS process, consists of a low-noise 20 kΩ trans-impedance amplifier (TIA), a 0 dB–28 dB four-step programmable gain amplifier (PGA), a 3:8 demultiplexer (DEMUX) and eight 3 mA–21 mA seven-step light emitting diode (LED) drivers. The receiver side consumes 2.4 mA from a 1.8 V supply, and the transmitter side draws up to 21 mA from an external 2 V supply to drive one of eight LEDs at a time. The total die area is 0.84 mm^2^.

This paper is organized as follows. In Section 2, we show the estimation process of the sugar content of apples using a tungsten halogen light source and a high-resolution NIR spectrometer. In Section 3, we find the minimum requirements for the design of an integrated NIR spectroscopy module, and in Section 4, we present the implemented NIR spectroscopy module with the simulated and measured results. Finally, the conclusion is given in Section 5.

## 2. Sugar Content Estimation Using an NIR Spectrometer

**Figure 1:**
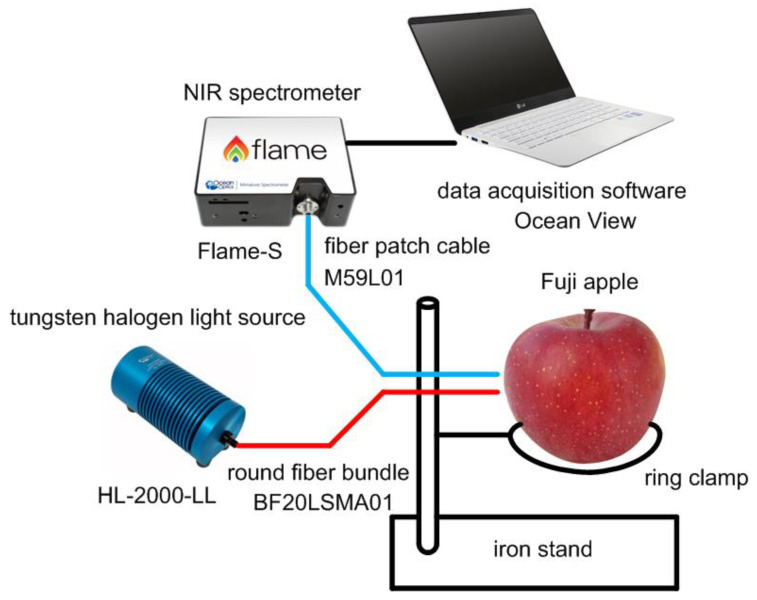
Sugar content measurement setup using an NIR spectrometer.

Figure 1 shows the sugar content measurement setup using an NIR spectrometer. We used a tungsten halogen light source (HL-2000-LL) and an NIR spectrometer (Flame-S), both from Ocean Insight, Inc (Geograaf 24 6921 EW Duiven, Nederland). [3]. Light from the source is sent to the surface of apples through a round fiber bundle (BF20LSMA01), which consists of 7 optical fibers with a 550 um core diameter, and diffuse reflected light from the apples is sent back to the NIR spectrometer through a fiber patch cable (M59L01), which is a single optical fiber with a 1 mm core diameter. Both are from Thorlabs, Inc (WG40530 and WG41050, Ely, UK). [4]. An apple is firmly placed on a ring clamp that is connected to an iron stand as shown in Figure 1.

The tungsten halogen light source emits visible (VIS) to NIR light over a broad range of 360 nm–2000 nm wavelengths, and the NIR spectrometer has a wavelength resolution of 0.2 nm and an ADC resolution of 16b. In this measurement, to reduce the necessity of processing redundant data, we utilized only 31 wavelengths, which were evenly spaced in increments of 10 nm over the range of 700 nm–1000 nm.

Since we acquired three samples from each apple and prepared 30 Fuji apples, the total number of samples was 90. The measurement was carried out at the room temperature of 20 °C in a dark room. The measured absorbance values were preprocessed by the standard normal variate transformation (SNV) and the first derivative [5,6]. By using multivariate linear regression (MLR) [7], a linear combination of the preprocessed absorbance values was used as an estimate of the sugar content of each sample. In this way, the indirectly estimated sugar content value was compared with the directly measured sugar content value obtained using a digital refractometer [8,9,10,11,12,13,14,15,16,17,18,19,20].

**Figure 2:**
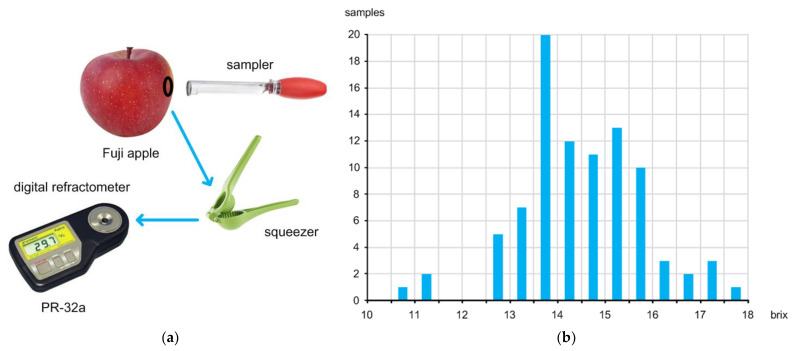
(**a**) Direct sugar content measurement setup using a digital refractometer and (**b**) distribution of measured sugar content values.

Figure 2a shows the direct sugar content measurement setup using a digital refractometer. To directly measure the sugar content, we should obtain a sample from each apple first by using a sampler. After squeezing each sample, we can obtain a couple of juice drops that are next placed on the lens of the digital refractometer (PR-32a) to measure the sugar content value. This digital refractometer can measure the sugar content value within an accuracy of ±0.1% [21].

**Figure 3:**
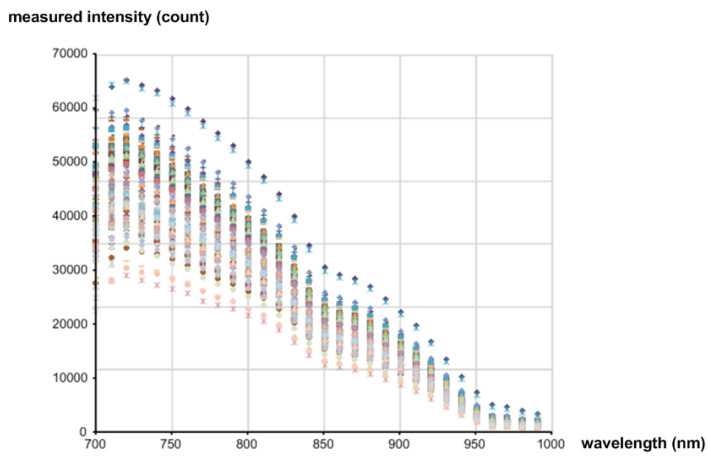
Measured intensities of diffuse reflected NIR light coming from all the samples.

**Figure 4:**
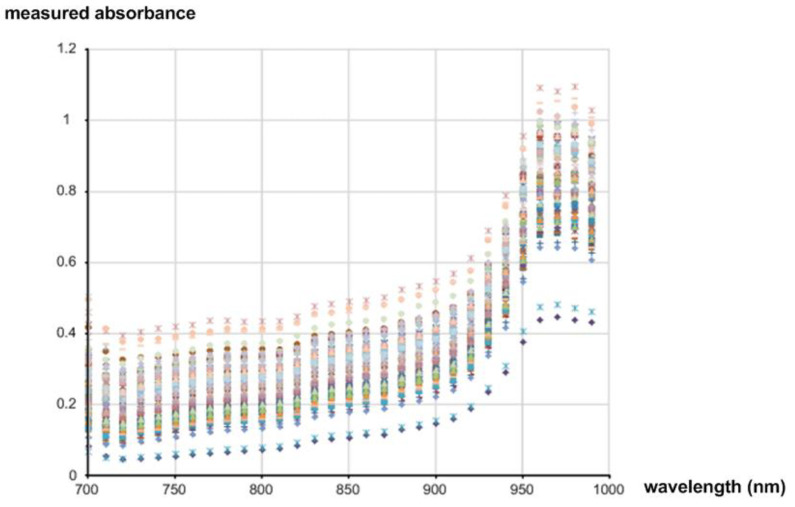
Measured absorbance values over 700 nm–1000 nm wavelengths.

Figure 2b shows the distribution of the measured sugar content values of the 90 samples. The directly measured sugar content values of apples are distributed over the range of 10 brix to 18 brix. The average is about 14.5 brix. Figure 3 shows the measured intensities of the diffuse reflected light coming from all the samples. Since the ADC resolution was 16b, the values were measured between 0 and 65,535 (2^16^ − 1). Figure 4 shows the measured absorbance values over 700 nm–1000 nm wavelengths, which were calculated by using the Lambert–Beer law [22]:

(1) $$A=log_{10}\frac{I_{0}-I_{dark}}{I-I_{dark}}=\varepsilon \times l\times c$$

where *A* is the absorbance, *ε* is the molar absorption coefficient, *l* is the path length, *c* is the concentration, *I_dark_* is the intensity of light without any light source, *I*_0_ is the intensity of light before passing through a sample, and *I* is the intensity of light after passing through a sample. To obtain *I*_0_, we measured the light intensity coming directly from the reflection standard that was made of polytetrafluoroethylene (PTFE), and to obtain *I_dark_*, we measured the light intensity while turning off all the light sources in a dark room.

**Figure 5:**
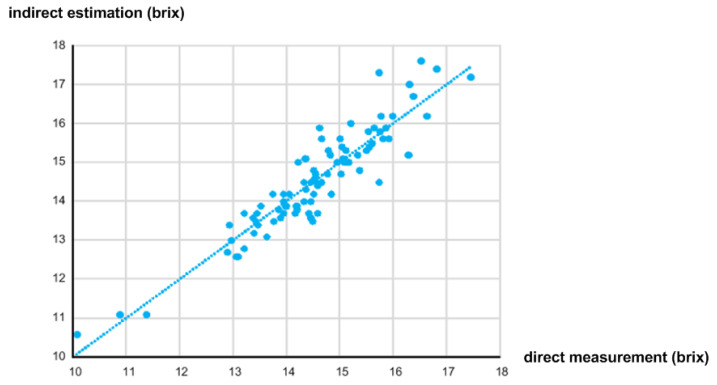
Correlation between indirect estimation and direct measurement of sugar content.

The correlation between the estimated sugar content value (*y* axis) using the NIR spectrometer and the measured sugar content value (*x* axis) using the digital refractometer is plotted in Figure 5. Since the correlation coefficient, *R*^2^, and the standard error of calibration (SEC) are defined as

(2) $$R^{2}=\frac{\sum_{i=1}^{n}{\left({\hat{y}}_{i}-\bar{y}\right)}^{2}}{\sum_{i=1}^{n}{\left(y_{i}-\bar{y}\right)}^{2}}$$

(3) $$SEC=\sqrt{\frac{\sum_{i=1}^{n}{\left(y_{i}-{\hat{y}}_{i}\right)}^{2}}{n-p-1}}$$

where $y_{i}$ is the estimated sugar content, ${\hat{y}}_{i}$ is the fitted value of $y_{i}$, $\bar{y}$ is the mean value of $y_{i}$, *n* is the number of samples, and *p* is the number of coefficients in the estimation model [7], they were calculated as 0.846 and 0.475 brix, respectively.

## 3. Minimum Requirements of Wavelength Number and ADC Resolution

If we use a tungsten halogen light source and an NIR spectrometer to estimate the sugar content value of apples, we can benefit from tens or hundreds of densely located wavelengths and a very high ADC resolution. However, since we aimed to implement an integrated NIR spectroscopy module, we had to determine the minimum number of wavelengths and the minimum ADC resolution while also not sacrificing the sugar content estimation accuracy too much.

**Figure 6:**
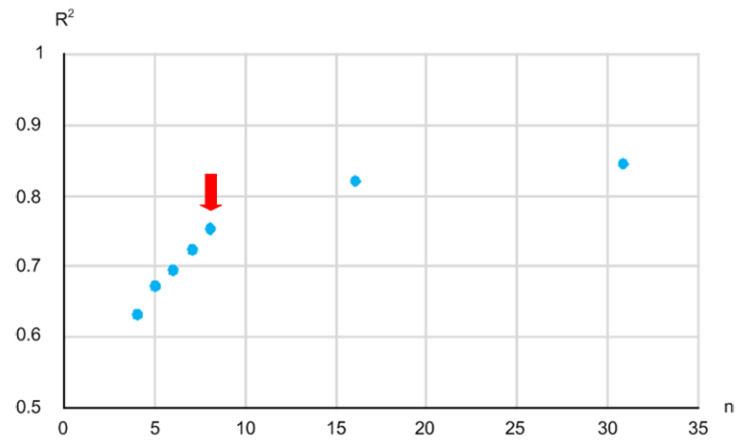
Relationship between the R^2^ and the number (*n*) of wavelengths.

For this goal, we estimated the sugar content value by using a linear combination of the absorbance values calculated from the measured light intensities at 31 different wavelengths that were evenly spaced in increments of 10 nm over the range of 700 nm to 1000 nm by adopting the MLR technique [7]. Then, we compared the R^2^ between the estimated sugar content value and the measured sugar content value while decreasing the number of wavelengths from 31 to 16, 8, 7, 6, and so on. Figure 6 shows the tendency of the measured R^2^ against the number of wavelengths. The R^2^ was measured as 0.846, 0.822, and 0.754 when *n* was 31, 16, and 8, respectively. It dropped abruptly when *n* decreased to less than 8. Thus, we determined the minimum required number of wavelengths to be 8, considering the hardware complexity of the implemented NIR spectroscopy module that should be mounted on a printed circuit board (PCB) with the same number of off-chip LEDs. The selected wavelengths were 720 nm, 750 nm, 780 nm, 810 nm, 840 nm, 870 nm, 910 nm, and 940 nm. In this paper, we selected only the wavelengths that were evenly distributed in increments of 30 nm. This was due to the restricted availability of the LEDs at different wavelengths. However, the performance of the NIR spectroscopy module could be improved if we select the optimum wavelengths by following the appropriate feature selection approach for the purpose of maximizing the R^2^ given a number of wavelengths.

**Figure 7:**
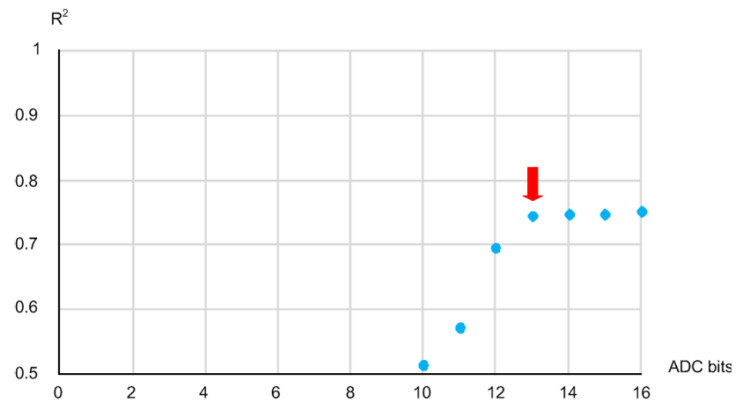
Relationship between the R^2^ and ADC resolution.

Then, we repeated the estimation process using the MLR technique again while decreasing the resolution of the diffuse reflected light intensities that were initially obtained using a 16b ADC of the NIR spectrometer 1b by 1b starting from 16b. We could reduce the ADC resolution by erasing the least significant bit (LSB) of the measured light intensities 1b by 1b at a time. Figure 7 shows the measured R^2^ versus the ADC resolution from 16b to 10b. Since the tendency of the measured R^2^ shows that the estimation accuracy degraded abruptly at the resolution of 12b, we determined the minimum required ADC resolution to be 13b as shown in Figure 7.

## 4. Design of an Integrated NIR Spectroscopy Module

**Figure 8:**
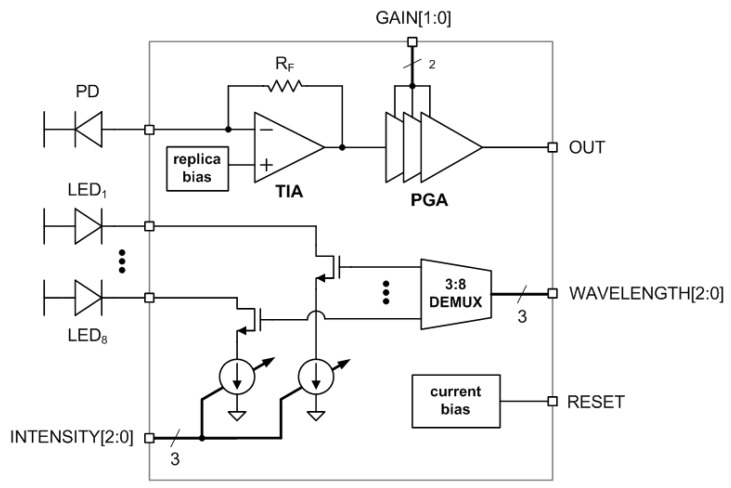
Architecture of the implemented NIR spectroscopy module.

Figure 8 shows the architecture of the implemented NIR spectroscopy module. The receiver side consists of an off-chip photo diode (PD), a low-noise 20 kΩ TIA, and a 0 dB–28 dB four-step PGA. The PD adopted in this work was Vishay Semiconductor’s TEMD5010 × 01 [23]. It has a sensing area of 7.5 mm^2^, and its dark current is typically 2 nA. Its spectral bandwidth is from 600 nm to 1040 nm, and there is typically a flow of a reverse light current of 55 uA for 1 mW/cm^2^ irradiance. Its input capacitance is about 25 pF. Meanwhile, the transmitter side consists of a 3:8 DEMUX and eight 3 mA–21 mA seven-step LED drivers. It can draw up to 21 mA from an external 2 V supply to drive one of eight LEDs at a time. The LEDs are Marubeni’s SMT models [24]. They have a radiant intensity of 6–20 mW/sr and a spectral bandwidth of about 20 nm. The maximum forward bias voltage is from 1.3 V to 1.7 V.

### 4.1. Trans-Impedance Amplifier

**Figure 9:**
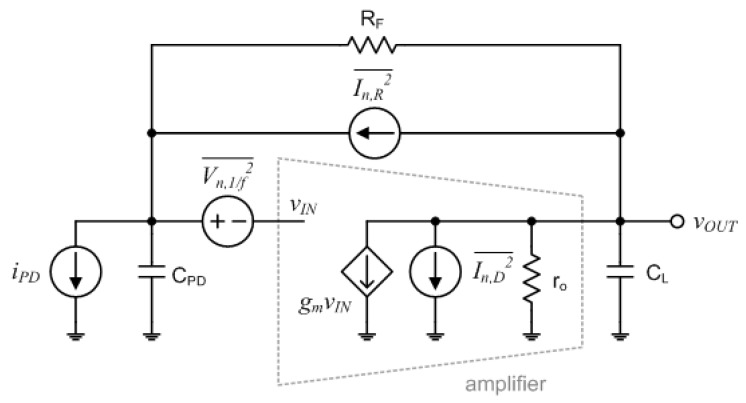
Simplified model of the trans-impedance amplifier for noise analysis.

The TIA converts an input current signal to an output DC voltage signal. Thus, in this NIR spectroscopy module, the performances of the gain, the output dynamic range, and the noise were of more interest rather than the bandwidth. Figure 9 shows a simplified model of the TIA for noise analysis. The feedback resistor, R_F_, which is placed between the input and output nodes, approximately determines the magnitude of the TIA gain. The TIA gain is expressed as

(4) $$Z_{T}=\frac{r_{o}\left(g_{m}R_{F}-1\right)}{1+g_{m}r_{o}}\times \frac{1}{1+s\frac{C_{PD}\left(r_{o}+R_{F}\right)+C_{L}r_{o}}{1+g_{m}r_{o}}+s^{2}\frac{C_{PD}C_{L}r_{o}R_{F}}{1+g_{m}r_{o}}}\approx R_{F}\times \frac{1}{1+\frac{s}{a}}\times \frac{1}{1+\frac{s}{b}}\;$$

where *a* and *b* are defined as

(5) $$a=\zeta \omega_{0}+\omega_{0}\sqrt{\zeta^{2}-1}$$

(6) $$b=\zeta \omega_{0}-\omega_{0}\sqrt{\zeta^{2}-1}$$

if we assume that $g_{m}r_{o}\gg 1$ and $g_{m}R_{F}\gg 1$. Here, *ω*_0_ and *ζ* are defined as follows:

(7) $$\omega_{0}=\sqrt{\frac{g_{m}}{C_{PD}C_{L}R_{F}}}$$

(8) $$\zeta =\frac{1}{2}\times \frac{C_{PD}r_{o}+C_{PD}R_{F}+C_{L}r_{o}}{\sqrt{g_{m}r_{o}{}^{2}C_{PD}C_{L}R_{F}}}$$

In addition, the equivalent spectral density of the input referred noise current of the TIA is expressed as follows [25,26,27,28,29]:

(9) $$\bar{I_{n,in}{}^{2}}=\frac{4kT}{R_{F}}+\frac{4kT\gamma }{g_{m}R_{F}{}^{2}}+\frac{4kT\gamma }{g_{m}}\omega^{2}C_{PD}{}^{2}+\frac{K}{C_{OX}WLR_{F}{}^{2}\omega }+\frac{K}{C_{OX}WL}\omega C_{PD}{}^{2}$$

Since the equivalent spectral density of the output referred noise voltage of the TIA can be obtained as

(10) $$\bar{V_{n,out}{}^{2}}=\bar{I_{n,in}{}^{2}}\times {\left|Z_{T}\right|}^{2}$$

taking into consideration the transfer function of (4), we can say that the mean square output noise voltage of the TIA decreases as the second pole of the transfer function decreases.

**Figure 10:**
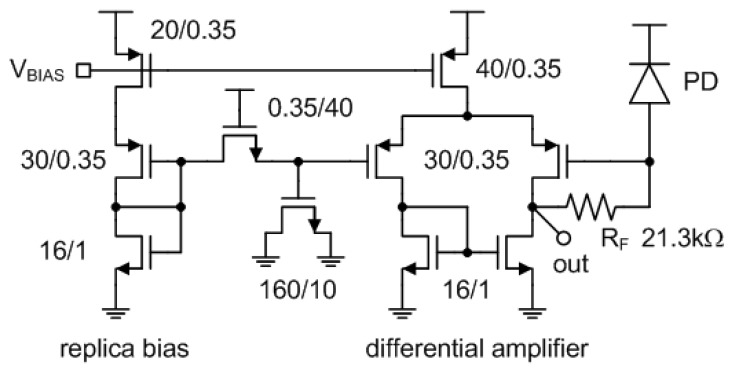
Schematic of the TIA.

**Figure 11:**
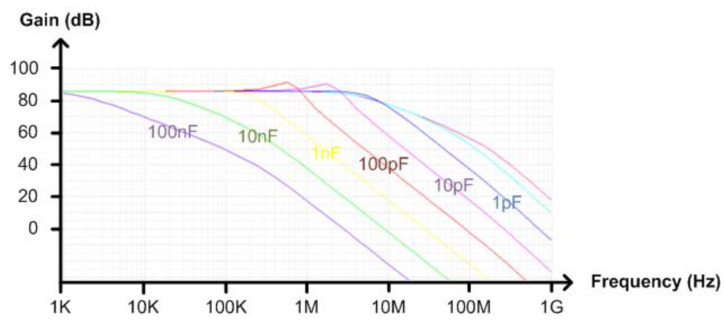
Simulated gain of the TIA versus C_L_.

**Figure 12:**
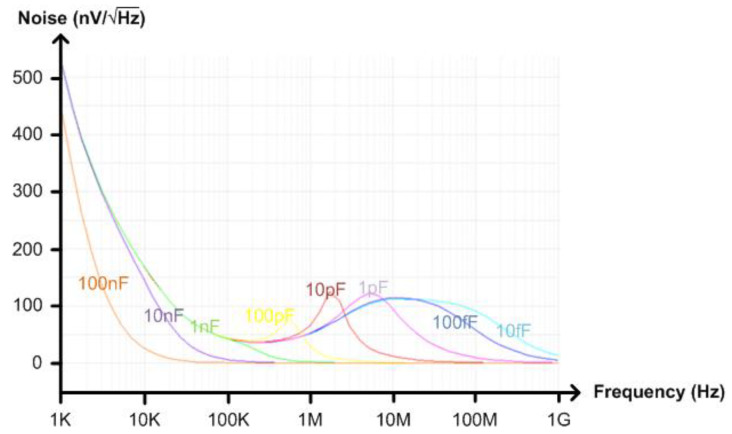
Simulated noise of the TIA versus C_L_.

Figure 10 shows the schematic of the implemented TIA. We used PMOS transistors instead of NMOS transistors in the input stage and the tail current source for reduced 1/f noise. The DC bias voltage applied to the positive input terminal of the differential amplifier comes from a replica bias circuit as shown in Figure 10. By using this replica bias, the input and output DC voltages of the TIA can be made equal to each other. To filter out the noise generated from the replica bias, we added the first order RC low-pass filter between the replica bias circuit and the differential amplifier. As the output referred noise voltage is directly affected by the transfer function of (4), it varies depending on C_L_. If C_L_ is set to be as large as 100 nF, the simulated bandwidth is less than 1 kHz and the simulated output referred noise voltage can be decreased to as low as 13 μV_rms_ as shown in Figure 11 and Figure 12, respectively. To guarantee a 13b ADC resolution at the output of the implemented NIR spectroscopy module, we made the peak-to-peak output dynamic range of the TIA larger than 300 mV. In case more than 13b ADC resolution is required by the measurement setup, the TIA gain should be increased or the output referred noise voltage should be decreased.

### 4.2. Programmable Gain Amplifier

The PGA amplifies the output DC voltage signal coming from the TIA with a variable voltage gain. Its main function is to enhance the output dynamic range for easy measurement in the next ADC stage. In this paper, the PGA is composed of cascaded three stages, and each stage has a binary switched voltage gain that is set to 0 dB or 9.4 dB. Thus, the total voltage gain can be controlled from 0 dB to 28.2 dB with a step size of 9.4 dB. Of course, we can more finely control the total voltage gain of the PGA with a smaller step size to enhance the output dynamic range. However, this will necessitate a greater number of stages and increase power consumption.

**Figure 13:**
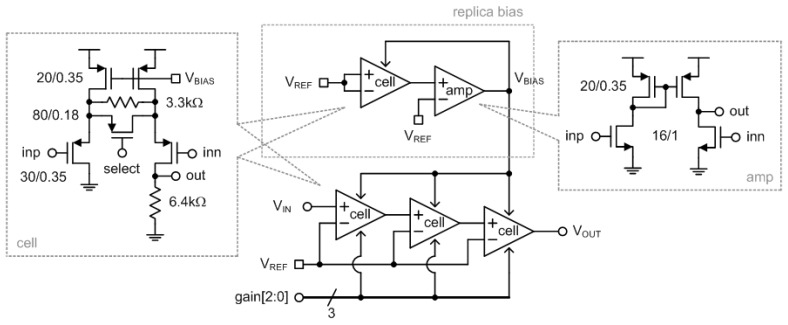
Architecture of the implemented PGA.

Figure 13 shows the architecture of the implemented PGA. Each PGA cell was designed with PMOS transistors to suitably process the signal from the previous TIA, which was also designed with PMOS transistors. The voltage gain of each PGA cell is determined by a 1b select signal. If the select signal is high, the gain is 0 dB, and if the select signal is low, the gain is 9.4 dB. The PMOS bias voltage, V_BIAS_, is generated from the replica bias circuit for the purpose of making the output voltage of the PGA cell inside the replica bias circuit be equal to V_REF_ when V_REF_ is applied to both inputs of the PGA cell, as shown in Figure 13. Then, if the generated V_BIAS_ is applied to three PGA cells, V_OUT_ will be equal to V_REF_ when V_REF_ is applied to V_IN_. In this design, V_REF_ was set as 700 mV. This PGA consumes 2 mA from a 1.2 V supply, and the maximum output dynamic range is 760 mV.

### 4.3. 3:8 DEMUX and 8 LED Drivers

The 3:8 DEMUX chooses one of eight LEDs at different wavelengths, and the LED driver flows a digitally controlled DC current through the chosen LED.

**Figure 14:**
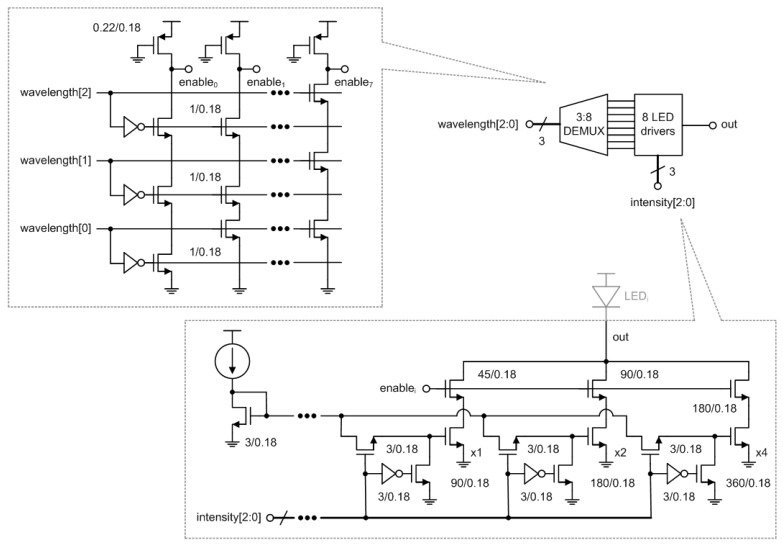
Architecture of the 3:8 DEMUX and 8 LED drivers.

Figure 14 shows the architecture of the 3:8 DEMUX and eight LED drivers. The 3:8 DEMUX was implemented in a pseudo NMOS logic and carries out binary to thermometer decoding to turn on one of eight enable signals based on a 3b digital control word, wavelength [2:0]. The LED driver appropriately switches on three NMOS current sources whose W/L ratios are scaled as x1, x2, and x4, respectively, according to another 3b digital control word, intensity [2:0]. Thus, the output DC current of the LED driver can be increased up to 21 mA with a step size of 3 mA. Among the eight LED drivers, only one whose enable signal is set to high drives the corresponding LED at a time.

### 4.4. Measurement

**Figure 15:**
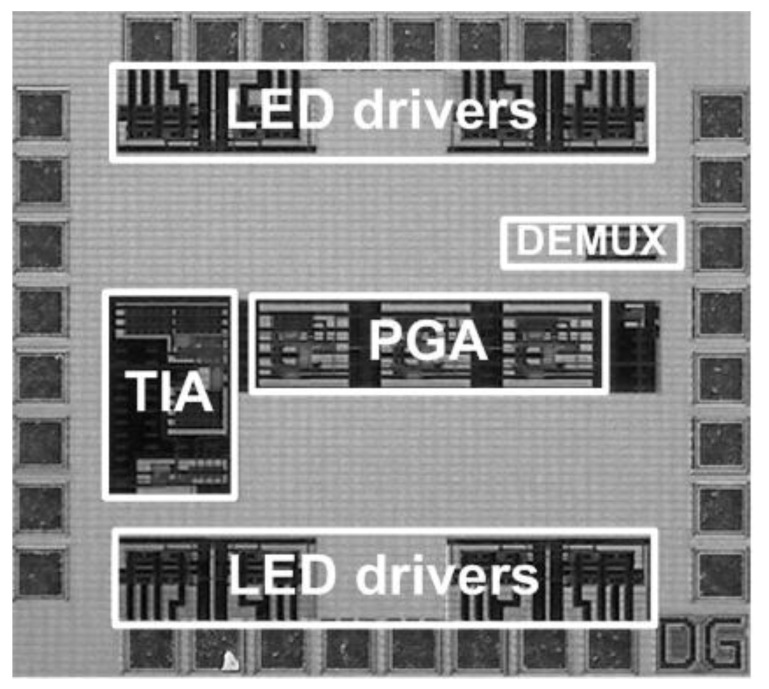
Die photo.

**Figure 16:**
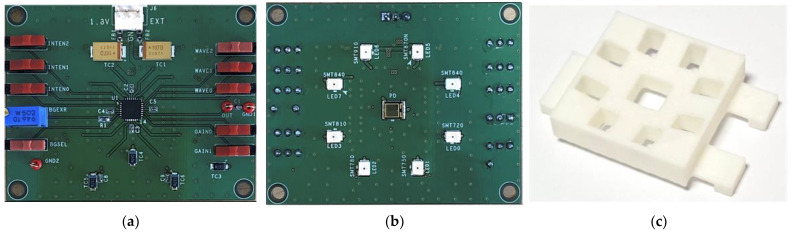
PCB (**a**) front face, (**b**) back face, and (**c**) chassis built by a 3D printer.

**Figure 17:**
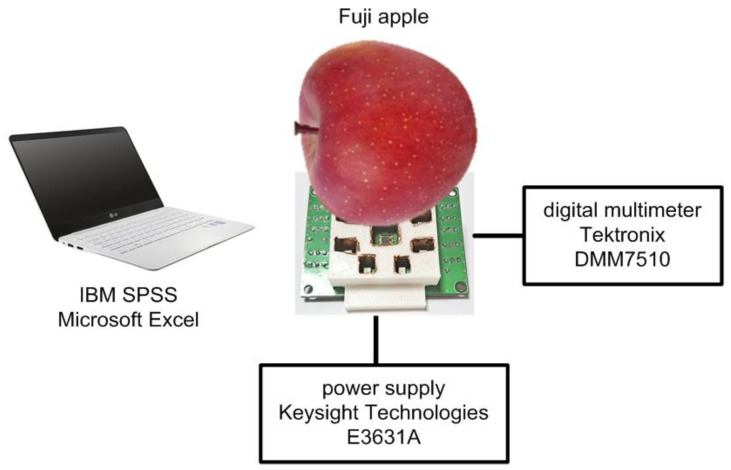
Sugar content measurement setup using the implemented NIR spectroscopy module.

An integrated NIR spectroscopy prototype module was implemented in a 0.18 μm 1P6M CMOS process. This module consists of a low-noise 20 kΩ TIA, a 0 dB–28 dB four-step PGA, a 3:8 DEMUX, and eight LED drivers. The receiver side consumes 2.4 mA from a 1.8 V supply, and the transmitter side consumes 21 mA from an external 2 V supply to drive one of eight LEDs alternately. The total die area is 0.84 mm^2^. Figure 15 shows the die photo, and Figure 16 shows the (a) front face and (b) back face of the four-layer FR-4 PCB on which the integrated NIR spectroscopy module packaged in a 28 pin micro lead frame (MLF) is mounted. On the front face of the PCB, the integrated NIR spectroscopy module is located with the supply connector and the several digital control switches, and on the back face of the PCB, there are eight LEDs surrounding the PD located at the center. Additionally, we built a chassis using a 3D printer [30], as shown in Figure 16c, to combine the flat-shaped PCB and the globular-shaped apples more closely. In addition, we attached a copper tape on the inside walls of the chassis to shield and protect the ambient light coming through from the LEDs to the PD directly. Figure 17 shows the sugar content measurement setup using the implemented NIR spectroscopy module. The wavelength and the intensity of LEDs were manually controlled in sequence using the on-board digital switches, and the DC output signal of the PGA was measured by using the high-resolution digital multi-meter.

**Figure 18:**
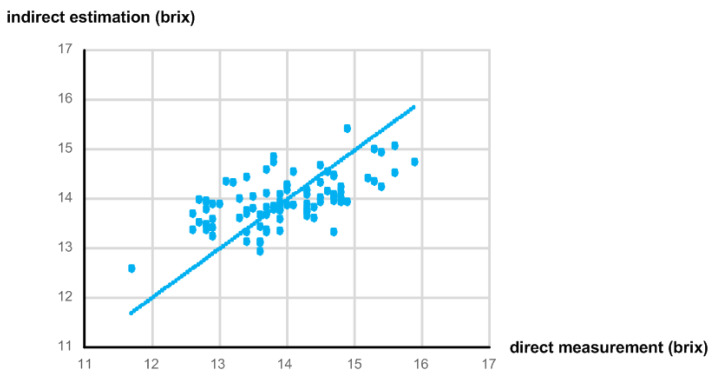
Correlation between indirect estimation and direct measurement of sugar content.

Figure 18 shows the correlation between the indirectly estimated sugar content value using the implemented NIR spectroscopy module and the directly measured sugar content value using the digital refractometer. Since we obtained three samples from each apple and prepared 30 Fuji apples, the total number of samples was 90. The sugar content was measured in a dark room at 20 °C. The PD, TEMD5010 × 01, from Vishay was used, and eight LEDs from Marubeni such as SMT720, SMT750, SMT780, SMT810, SMT840, SMT870N, SMT910, and SMT940 were used. These PD and LEDs were placed on the PCB about 15 mm apart from each other. The measured R^2^ and SEC were 0.365 and 0.686 brix, respectively. Compared to Figure 5, the correlation was somewhat degraded since the integrated NIR spectroscopy module utilized less number of wavelengths and a lower ADC resolution. Nevertheless, if we determine how to solve the problems of the different light paths between the PD and eight LEDs and the variant wavelengths and intensities of eight LEDs, we expect the integrated NIR spectroscopy module to show better performance.

## 5. Conclusions

In this paper, an integrated NIR spectroscopy prototype module was implemented in a 0.18 μm 1P6M CMOS process. To design an integrated NIR spectroscopy module, we first examined the sugar content estimation process using an NIR spectrometer with a 10 nm wavelength resolution and a 16b ADC resolution, and then we carefully observed the estimation accuracy while decreasing the number of wavelengths and the ADC resolution. Finally, we chose eight wavelengths and a 13b ADC resolution. The implemented NIR spectroscopy module occupies a small die area of 0.84 mm^2^ and estimated the sugar content value with the measured R^2^ and SEC to be 0.365 and 0.686 brix, respectively.

## Data Availability

Not applicable.

## References

[1] Campeanu G., Neata G., Darjanschi G. (2006). Chemical composition of the fruits of several apple cultivars growth as biological crop. Not. Bot. Horti Agrobot. Cluj-Napoca.

[2] Ackermann J., Fischer M., Amado R. (1992). Changes in sugars, acids, and amino acids during ripening and storage of apples. J. Agric. Food Chem..

[3] Ocean Insight Home Page. http://oceaninsight.com.

[4] Thorlabs Home Page. http://thorlabs.com.

[5] Ozaki Y., McClure W.F., Christy A.A. (2007). Near-Infrared Spectroscopy in Food Science and Technology.

[6] Siesler H.W., Ozaki Y., Kawata S., Heise H.M. (2002). Near-Infrared Spectroscopy.

[7] Chatterjee S., Hadi A.S. (2012). Regression Analysis by Example.

[8] Tsuta M., Sugiyama J., Sagara Y. (2002). Near-infrared imaging spectroscopy based on sugar absorption band for melons. J. Agric. Food Chem..

[9] Wang H., Peng J., Xie C., Bao Y., He Y. (2015). Fruit quality evaluation using spectroscopy technology: A review. Sensors.

[10] Ozaki Y. (2012). Near-infrared spectroscopy-its versatility in analytical chemistry. Anal. Sci..

[11] Lee Y., Han S.-H. (2016). Feasibility of nondestructive sugar content analysis of Korean pears by using near-infrared diffuse-reflectance spectroscopy. Bull. Korean Chem. Soc..

[12] Kawano S., Watanabe H., Iwamoto M. (1992). Determination of sugar content in intact peaches by near infrared spectroscopy. J. Jpn. Soc. Hortic. Sci..

[13] Golic M., Walsh K., Lawson P. (2003). Short-wavelength near-infrared spectra of sucrose, glucose, and fructose with respect to sugar concentration and temperature. Appl. Spectrosc..

[14] Nicolai B.M., Beullens K., Bobelyn E., Peirs A., Saeys W., Theron K.I., Lammertyn J. (2007). Nondestructive measurement of fruit and vegerable quality by means of NIR spectroscopy: A review. Postharvest Biol. Technol..

[15] Valavani A., Chowdhury S., Burnett A.D., Clarkson A.R., Bacon D.R., Khanna S.P., Davies A.G., Linfield E.H., Dean P. (2016). Diffuse-reflection spectroscopy using a frequency-switchable terahertz quantum cascade laser. IEEE Trans. Terahertz Sci. Technol..

[16] Tian G., Li X., Zhang B., Zhou J., Gu B. (2019). Comparative study of two different strategies for determination of soluble solids content of apples from multiple geographical regions by using FT-NIR spectroscopy. IEEE Access.

[17] Li X., Bi S., Zhang Y., Shen T. SVM-based apple classification of soluble solids content by near-infrared spectroscopy. Proceedings of the Chines Control and Decision Conference (CCDC).

[18] Shen Y., Wu Y., Li L., Li L. Nondestructive detection for forecasting the level of acidity and sweetness of apple based on NIR spectroscopy. Proceedings of the IEEE 2nd Advanced Information Technology, Electronic and Automation Control Conference (IAEAC).

[19] Abasi S., Minaei S., Jamshidi B., Fathi D. (2021). Development of an optical smart portable instrument for fruit quality detection. IEEE Trans. Instrum. Meas..

[20] Tran N.-T., Phan Q.-T., Nguyen C.-N., Fukuzawa M. Machine learning-based classification of apple sweetness with multispectral sensor. Proceedings of the 21st ACIS International Winter Conference on Software Engineering, Artificial Intelligence, Networking and Parallel/Distributed Computing (SNPD-Winter).

[21] Specification Document of PR-32a, Atago Co., Ltd. http://atago.net/en/products-palette-top.php.

[22] Swinehart D.F. (1962). The Beer-Lambert Law. J. Chem. Educ..

[23] Datasheet of TEMD5010X01, Vishay Semiconductors. http://www.vishay.com.

[24] Datasheets of SMT Type LEDs, Marubeni Inc. http://www.marubeni.com.

[25] Sackinger E. (2017). Analysis and Design of Transimpedance Amplifiers for Optical Receivers.

[26] Ahmed M.G., Talegaonkar M., Elkholy A., Shu G., Elmallah A., Rylyakov A., Hanumolu P.K. (2018). A 12-Gb/s–16.8-dBm OMA sensitivity 23mW optical receiver in 65-nm CMOS. IEEE J. Solid-State Circuits.

[27] Johns D., Markin K. (1996). Analog Integrated Circuit Design.

[28] Kumar V., Saravanan S., Duraiswamy P., Selvaraja S.K. (2021). Single stage low noise inductor-less TIA for RF over fibre communication. IEEE Access.

[29] Ying D., Hall D.A. (2021). Current sensing front-ends: A review and design guidance. IEEE Sens. J..

[30] Datasheet of 3DWOX DP201, Sindoh. http://3dprinter.sindoh.com.

